# Toxicological impact of Thiamethoxam on adult male rats: Histopathological, biochemical, and oxidative DNA damage assessment

**DOI:** 10.1016/j.toxrep.2025.101983

**Published:** 2025-03-15

**Authors:** Sahar Y. Issa, S.M. Abdel Rahman, Yasmin M. Gaber, Nada A.H. Soliman

**Affiliations:** a Department of Forensic Medicine and Clinical Toxicology. Faculty of Medicine - Alexandria University, Egypt; b Central Laboratory, Alexandria University, Egypt; c Medical Intern, Faculty of Medicine, Alexandria University, Egypt; d Department of Medical Biochemistry, Faculty of Medicine, Alexandria University, Egypt

**Keywords:** Thiamethoxam, Pesticide, Toxicity, Neonicotinoids, Biochemical, hepatotoxicity, nephrotoxicity, testicular toxicity

## Abstract

**Background:**

Thiamethoxam (TMX), a widely used second-generation neonicotinoid insecticide, has raised concerns due to its toxic effects on non-target species, including mammals. Its prolonged use is associated with hepatotoxicity, nephrotoxicity, and reproductive damage.

**Objectives:**

This study evaluates the dose-dependent biochemical, histopathological, and genetic toxic effects of TMX in male albino rats, emphasizing its impact on the liver, kidney, and reproductive systems.

**Materials and methods:**

Forty male Wistar albino rats were assigned to control and three experimental groups treated with TMX at 26, 39, and 78 mg/kg/day over eight weeks. Key biochemical markers such as Alanine transaminase (ALT), Aspartate transaminase (AST), urea, creatinine and oxidative stress indicators (Catalase (CAT), Glutathione (GSH), Malondialdehyde (MDA), and reproductive parameters (testosterone, sperm count, and motility) were analyzed. Histopathological examination of the liver, kidney, and testes was performed, alongside evaluation of Deoxyribonucleic Acid (DNA) damage in testicular tissue.

**Results:**

TMX exposure caused significant dose-dependent increases in liver and kidney function markers and oxidative stress. Reproductive toxicity was evident, with reduced testosterone levels, impaired sperm parameters, and histopathological damage to testicular tissue. Notably, TMX induced oxidative DNA damage in testicular tissue, as indicated by increased levels of 8-hydroxy-2′-deoxyguanosine.

**Conclusions:**

This study highlights TMX's systemic toxicity in a dose-dependent manner, with oxidative stress and DNA damage as key mechanisms. The findings underscore the need for stricter regulatory measures and further exploration of protective strategies to mitigate TMX-induced toxicity.

## Highlights

• Thiamethoxam caused dose-dependent disruptions in biochemical markers and oxidative stress.
• It led to sperm abnormalities, reduced testosterone, and DNA damage.
• Severe liver, kidney, and testicular damage occurred at higher doses.

## Graphical Abstract

**Figure:**
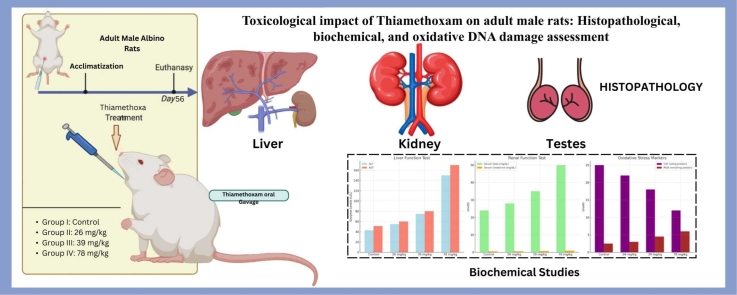

## 1 Introduction

The widespread pesticide use in modern agriculture has raised growing concerns regarding its toxic consequences on non-target organisms, such as humans [16]. Among these, neonicotinoids have gained prominence due to their targeted action on insect nicotinic acetylcholine receptors (nAChRs) and their perceived lower toxicity to mammals. Thiamethoxam (TMX), a second-generation neonicotinoid, is extensively utilized for pest management across diverse crops. However, emerging evidence suggests that TMX may exert significant toxicological effects beyond its intended targets, mainly when metabolized into compounds interacting with mammalian nAChRs [22], [15].

While TMX was designed to minimize toxicity in vertebrates, studies have reported its adverse impacts on mammalian systems, including oxidative stress, organ dysfunction, and disruption of reproductive health. TMX-induced oxidative stress has been implicated in hepatotoxicity, nephrotoxicity, and testicular damage [2], [1].

Rodrigues et al. reported that exposure to the neonicotinoid insecticide can lead to changes in the cholinergic system of rats. These changes were associated with both biochemical and behavioural effects, which may resemble the toxic impacts of other pesticides known to contribute to the development of neurodegenerative disorders, including Alzheimer 's-like dementia [17]. Neonicotinoids are divided into three generations, with thiamethoxam, a member of the thianicotinyl subclass, being part of the second generation of neonicotinoids [11].

The liver, a primary site for detoxification and metabolism of xenobiotics, is particularly susceptible to pesticide-induced injury. Hepatotoxic effects, including alterations in enzyme activity and histopathological changes, have been observed following exposure to neonicotinoids [4]. Similarly, the kidneys, responsible for excreting metabolic waste and maintaining electrolyte balance, are vulnerable to nephrotoxicity caused by prolonged pesticide exposure [14]. Moreover, the reproductive system—due to its sensitivity to oxidative stress and endocrine disruption—emerges as a critical target for thiamethoxam-induced damage, with adverse effects reported on sperm quality, hormone levels, and testicular histology [9].

Research suggests that antioxidant mechanisms play a crucial role in mitigating TMX toxicity. According to Zuscikova et al. [24], oxidative stress is a major pathway of TMX-induced toxicity, and certain biological systems may have the potential to recover from damage through endogenous antioxidant responses and external antioxidant supplementation [24]. Additionally, studies on selenium and boron-based compounds have shown their potential in alleviating TMX-induced oxidative stress in various tissues, indicating that supportive interventions may enhance reversibility [23], [21].

Furthermore, research conducted on aquatic organisms and rodents has demonstrated that TMX exposure leads to oxidative damage and inflammatory responses, but partial recovery has been observed upon cessation of exposure and activation of detoxification pathways [7], [19]. However, the extent of recovery varies based on exposure duration, species, and tissue type.

This study tackles a critical literature gap by integrating biochemical, histopathological, and genetic analyses to evaluate TMX's systemic toxicity in male albino rats. Unlike previous research, which often focuses on single endpoints, this study comprehensively examines oxidative stress markers, DNA damage, and organ-specific toxicities, providing a holistic understanding of TMX's toxicological profile. The novel focus on TMX-induced genetic damage, assessed through oxidative DNA damage markers, represents a significant advancement in understanding the mechanisms underlying its reproductive and systemic toxicity.

## 2 Aims of the current work

Combining biochemical assays, oxidative stress markers, genetic toxicity, and histopathological analyses, this research aims to assess thiamethoxam's impact on the liver, kidneys, and testes in adult male albino rats.

## 3 Materials and methods

### 3.1 Animals

This study was done on 40 adult male Wistar Albino rats between 230 and 250 g weight. The rats were housed in groups of five per plastic cage, with eight cages, and kept under controlled laboratory conditions: a temperature of 25°C, a 12-hour light/dark cycle, and humidity levels between 65–75 %. They were provided with a balanced rat diet (60 % maize, 20 % soybean, 3 % molasses, 1.5 % bran, 0.5 % salt, and 0.2 % vitamins) purchased from the Agriculture Ministry's Animal Food Manufacturing Facility, along with unlimited access to water. The animals were given a two-week acclimatization period before the experiment began. All experimental procedures adhered to the guidelines outlined by the Ethical Institutional Review Board of Alexandria University, Egypt. This study followed the principles of the 1964 Declaration of Helsinki and its subsequent amendments, ensuring the humane treatment of animals. The protocols were approved under review number (09−03−22), confirming compliance with international standards for animal research, including provisions for minimizing pain and distress. At the end of the study, animals were euthanized humanely using intraperitoneal administration of sodium pentobarbital to ensure a painless and stress-free procedure.

### 3.2 Materials

The insecticide Thiamethoxam, produced by Syngenta Company Egypt, was purchased from the Egyptian Company for Seeds and Agricultural Chemicals. It contains 250 g/kg of the active ingredient Thiamethoxam (3-(2-chloro-thiazol-5-ylmethyl)-5-methyl-(1,3,5)-oxadiazinan-4-yldene-N-nitroamine) and 750 g/kg of inactive ingredients. Sigma-Aldrich Chemical Co. (St. Louis, MO, USA) was the source of all other chemicals used in the study. All the doses used in the experimental research are based on the active ingredient content of the commercial product. The commercial formulation’s concentration was used as specified by the manufacturer. However, to ensure accuracy, we conducted independent quantification of TMX using Gas Chromatography-Mass Spectrometry (GC-MS) (Agilent 7890B GC with 5977B Mass Selective Detector) in the central laboratory. This step confirmed the actual concentration, ensuring precise dosing and exposure assessment.

### 3.3 Experimental design

The selected doses of TMX (26, 39, and 78 mg/kg/day) were determined based on prior LD50 studies in rats, which identified an oral LD50 of 1563 mg/kg body weight [13], [20]. To simulate subchronic exposure scenarios, the low, medium, and high doses represent 1/60th, 1/40th, and 1/20th of the LD50 were used. These dosages were chosen to reflect potential human exposure levels in agricultural settings or environmental contamination scenarios where low-level chronic exposure occurs. This approach ensures relevance to both occupational and environmental health risk assessments.

Crushed and water-dissolved TMX was fed to rats via oral gavage. The rats were divided into four groups, each with ten rats. The experiment continued for eight weeks (56 days), corresponding to the completion of spermatogenesis. The rats were divided into four groups (I-IV): distilled water control group, TMX oral doses of 26, 39, and 78 mg/kg BW (1/60, 1/40, and 1/20 oral LD_50_) simultaneously.

### 3.4 Sample collection

At the end of the treatment period, the animals were fasted all night with free access to water and then euthanized using intraperitoneal injection of sodium pentobarbital. Blood samples (2 mL) were collected via cardiac puncture in non-heparinized tubes, centrifuged at 1500 × over 10 minutes using an Eppendorf 5810 R bench centrifuge, and the serum was stored at −20°C for later analysis. The liver, kidneys, and testes were promptly removed, weighed, and fixed in 10 % buffered formalin for histopathological studies. A portion of the liver (20 mg) from the anterior right lobe was excised and placed into ice-cold microcentrifuge tubes containing 1 mL of high-grade HPLC-grade water. The hepatic and renal samples were homogenized and centrifuged at 25,000 rpm for 20 minutes, and the supernatant was stored at −80°C for later analysis. The carefully dissected fat-free reproductive organs (testes, epididymis, prostate gland, and seminal vesicles) were washed, dried, and weighed. One testicle per rat was stored at −80°C for biochemical and oxidative stress-related analytical tests. The remaining testes and epididymis fixed in 10 % buffered formalin were later processed for histopathological examination.

E. Antioxidant enzyme quantification: The collected tissues were processed to measure oxidative stress markers and antioxidant enzymes. An automatic homogenizer was used to homogenize the tissues in phosphate-buffered saline (PBS), then centrifuged at 30,000 rpm for 30 minutes. The resulting supernatant was used for hormonal analysis, protein estimation, and antioxidant enzyme assays.

- Catalase (CAT) Colorimeter Assay: By measuring the change in absorbance brought on by H2O₂ breakdown, the approach outlined by Afsar et al. was used to estimate catalase activity. Two millilitres of phosphate buffer (pH 7.0) were used to dilute a 50 μL tissue homogenate sample. The absorbance was measured at 240 nm at intervals of 15 and 30 seconds following mixing. A change in absorbance of 0.01 per minute was considered one unit of CAT activity [3].

- Glutathione peroxidase (GSH) Colorimeter Assay: The technique created by Afsar et al. was followed. At 560 nm, the chromogen produced during the reaction was measured. Units per milligram of protein were used to express the results [3].

F. Lipid peroxidation Spectrophotometric Assay: Malondialdehyde (MDA) was assessed using the Hayashi et al. technique. 140 μL of sodium acetate buffer (pH 4.8) was combined with 5 mL H₂O₂ standards, and homogenate samples in 96-well plates for this experiment, which was then incubated for five minutes at 37°C. Each well was then filled with 100 μL of a solution of ferrous sulfate (1:25 ratio) and N, N′-diethyl-1,4-phenylenediamine (DEPPD), then incubated for one minute at 37°C. A microplate reader was used to measure absorbance at 505 nm, and readings were taken every 15 seconds for three minutes [10].

G. Hormone analysis: Testosterone levels were determined using a commercially available enzyme immunoassay (EIA) kit (Abcam, UK, Catalogue No. ab108666). The assay was conducted following the protocol provided by the manufacturer. Absorbance readings were taken at 450 nm using a microplate reader. The kit's detection sensitivity was 0.03 ng/mL, as specified in the product documentation.

H. Biochemical analysis: Serum levels of liver enzymes (ALT, AST, GGT, CK, ALP) were measured using EIA standard kits (Sigma-Aldrich, St. Louis, MO). Serum albumin levels were also measured to assess liver function. To assess kidney function, serum urea and creatinine levels were quantified.

I. DNA toxicity assessment: DNA damage was evaluated in testicular tissues using the 8-hydroxy-2′-deoxyguanosine (8-OH-2DG) assay, a well-established biomarker of oxidative DNA damage. Tissue homogenates were prepared from frozen testicular samples, and DNA was extracted using the phenol-chloroform method. The quantification of 8-OH-2DG levels was performed using an ELISA-based kit (Catalogue No. ab201734, Supplier: Abcam). This kit has a detection sensitivity of 0.1 ng/mL and high specificity and sensitivity for detecting oxidative modifications in nucleic acids, ensuring reliable genetic toxicity assessment.

### 3.5 Statistical analysis

The Statistical Package for Social Sciences (SPSS/version 24) program was used for the statistical analysis. Standard deviation (SD) ± mean was used to express the data. A one-way ANOVA was conducted to determine statistical significance (P). F-ratio (F) was calculated to show the differences between groups compared to the differences within each group.

## 4 Results

This study aimed to assess the effects of TMX on various physiological and biochemical markers across different groups of rats with varying doses. The effects of thiamethoxam (TMX) on liver function parameters are summarized in Table 1. TMX exposure caused dose-dependent increases in liver enzymes (ALT, AST, GGT) and other markers. The F-values indicate significant differences between groups in ALT, AST, GGT, CK, and ALP, with p-values < 0.0001, suggesting a highly significant impact of dosage on these parameters. The Albumin levels did not show statistical significance between the control and Group 2 (26 mg/kg),

**Table 1:** ANOVA statistical analysis of liver function tests in control and Thiamethoxam-treated groups.

Parameter	Control (Group 1)	Group 2 (26 mg/kg)	Group 3 (39 mg/kg)	Group 4 (78 mg/kg)	F	P
ALT (U/L)	43 ± 5	55 ± 7^a^	75 ± 10^b^	150 ± 20^c^	567.60	< 0.0001
AST (U/L)	51 ± 4	60 ± 8^a^	80 ± 12^b^	170 ± 20^c^	539.15	< 0.0001
GGT (U/L)	35 ± 3	40 ± 4^a^	55 ± 6^b^	100 ± 10^c^	1059.88	< 0.0001
CK (U/L)	81 ± 5	85 ± 6^a^	95 ± 10^b^	120 ± 12^c^	96.85	< 0.0001
ALP (U/L)	58 ± 5	65 ± 8^a^	75 ± 12^b^	110 ± 15^c^	179.52	< 0.0001
Albumin (g/dL)	3.4 ± 0.1	3.4 ± 0.1 ^ns^	3.1 ± 0.1^b^	2.8 ± 0.2^c^	-	-

- A one-way ANOVA was conducted to determine statistical significance (P).

- F-ratio (F) was calculated to show the differences between groups compared to the differences within each group

-N in all groups = 10

- Superscript meanings:

(a): Statistically significant at p ≤ 0.05

(b): Statistically significant at p ≤ 0.02–0.04

(c): Statistically significant at p < 0.001

(ns): Not statistically significant

Renal function parameters, presented in Table 2, indicated a dose-dependent increase in serum urea and creatinine levels. The F-values indicate significant differences among groups for both serum urea and serum creatinine, with p-values < 0.0001, suggesting that Thiamethoxam exposure significantly affects renal function. Serum urea levels increased in a dose-dependent manner, with the highest dose (78 mg/kg) showing the most significant rise. Serum creatinine also increased progressively with dosage, indicating potential impairment of renal function.

**Table 2:** ANOVA statistical analysis of renal function tests in control and Thiamethoxam-treated groups.

Parameter	Control (Group 1)	Group 2 (26 mg/kg)	Group 3 (39 mg/kg)	Group 4 (78 mg/kg)	F-value	P-value
Serum Urea (mg/dL)	24 ± 2	28 ± 3^a^	35 ± 5^b^	50 ± 8^c^	180.84	< 0.0001
Serum Creatinine (mg/dL)	0.5 ± 0.1	0.55 ± 0.1^a^	0.65 ± 0.1^b^	0.9 ± 0.15^c^	70.53	< 0.0001

- A one-way ANOVA was conducted to determine statistical significance (P).

- F-ratio (F) was calculated to show the differences between groups compared to the differences within each group

-N in all groups = 10

- Superscript meanings:

(a): Statistically significant at p ≤ 0.05

(b): Statistically significant at p ≤ 0.02–0.04

(c): Statistically significant at p < 0.001

(ns): Not statistically significant

The statistical significance (denoted by a, b, c) confirms that each group is significantly different from the control and preceding dosage groups.

The impact of TMX on body and organ weights is presented in Table 3. TMX exposure caused a dose-dependent reduction in body weight gain and organ weight. Liver and kidney weights were significantly lower in Group 4 (7.20 ± 0.30 g and 0.85 ± 0.04 g, respectively). Reproductive organs, including the testis (0.85 ± 0.05 g), prostate gland (0.10 ± 0.01 g), seminal vesicle (0.30 ± 0.07 g), and epididymis (0.35 ± 0.03 g), also showed substantial reductions in weight at the highest TMX dose. The F-values show highly significant differences between control and treated groups across all parameters.

**Table 3:** ANOVA statistical analysis of body and organ weights in control and Thiamethoxam-treated groups.

Parameter	Control (Group 1)	Group 2 (26 mg/kg)	Group 3 (39 mg/kg)	Group 4 (78 mg/kg)	F-value	P-value
Body Weight Gain (g)	192 ± 8	180 ± 7^a^	170 ± 9^b^	150 ± 10^c^	181.94	< 0.0001
Liver Weight (g)	8.20 ± 0.15	7.90 ± 0.20^a^	7.60 ± 0.25^b^	7.20 ± 0.30^c^	109.87	< 0.0001
Kidney Weight (g)	1.00 ± 0.05	0.95 ± 0.04^a^	0.90 ± 0.03^b^	0.85 ± 0.04^c^	71.33	< 0.0001
Testis Paired Weight (g)	1.20 ± 0.10	1.10 ± 0.08^a^	1.00 ± 0.06^b^	0.85 ± 0.05^c^	121.70	< 0.0001
Prostate Gland (g)	0.15 ± 0.02	0.13 ± 0.01^a^	0.12 ± 0.02^b^	0.10 ± 0.01^c^	44.19	< 0.0001

- A one-way ANOVA was conducted to determine statistical significance (P).

- F-ratio (F) was calculated to show the differences between groups compared to the differences within each group

-N in all groups = 10

- Superscript meanings:

(a): Statistically significant at p ≤ 0.05

(b): Statistically significant at p ≤ 0.02–0.04

(c): Statistically significant at p < 0.001

(ns): Not statistically significant

The paired testis weight, prostate gland weight, and seminal vesicle weight were significantly reduced in the treated groups, implying potential reproductive toxicity. The epididymis weight followed a similar trend, further indicating adverse effects on the male reproductive system.

As shown in Table 4, TMX exposure adversely affected reproductive parameters in a dose-dependent manner. Testosterone levels significantly decreased from 0.45 ± 0.05 ng/dL in the control to 0.22 ± 0.03 ng/dL in Group 4. Sperm count, viability, motility, motility progression, and fructose levels also declined across the treatment groups, with the most pronounced reductions observed in Group 4. The F-values indicate highly significant differences among groups for all reproductive parameters, with p-values < 0.0001.

**Table 4:** ANOVA statistical analysis of reproductive parameters after thiamethoxam exposure.

Parameter	Control (Group 1)	Group 2 (26 mg/kg)	Group 3 (39 mg/kg)	Group 4 (78 mg/kg)	F-value	P-value
Testosterone (ng/dL)	0.45 ± 0.05	0.35 ± 0.03^a^	0.28 ± 0.04^b^	0.22 ± 0.03^c^	210.08	< 0.0001
Sperm Count (million/mL)	80 ± 5	75 ± 6^a^	65 ± 7^b^	50 ± 8^c^	115.51	< 0.0001
Percent Viability (%)	85 ± 3	80 ± 4^a^	70 ± 5^b^	55 ± 6^c^	393.75	< 0.0001
Motility (%)	75 ± 4	70 ± 5^a^	60 ± 6^b^	45 ± 7^c^	142.86	< 0.0001
Motility Progression (%)	70 ± 5	65 ± 4^a^	55 ± 5^b^	40 ± 6^c^	205.40	< 0.0001
Fructose Levels (mg/dL)	180 ± 10	170 ± 12^a^	150 ± 15^b^	120 ± 20^c^	-	-

- A one-way ANOVA was conducted to determine statistical significance (P).

- F-ratio (F) was calculated to show the differences between groups compared to the differences within each group

-N in all groups = 10

- Superscript meanings:

(a): Statistically significant at p ≤ 0.05

(b): Statistically significant at p ≤ 0.02–0.04

(c): Statistically significant at p < 0.001

(ns): Not statistically significant

The levels of oxidative stress markers are presented in Table 5. TMX exposure caused a significant decrease in antioxidant enzyme activities (CAT and GSH) and increased MDA levels, reflecting oxidative damage. Group 4 exhibited the lowest CAT (12.0 ± 1.5 U/mg protein) and GSH (20.0 ± 1.8 nmol/mg protein) levels, alongside the highest MDA levels (6.0 ± 0.6 nmol/mg protein). The F-values show highly significant differences between control and treated groups for all oxidative stress markers, with p-values < 0.0001.

**Table 5:** ANOVA statistical analysis of oxidative stress markers after thiamethoxam exposure.

Parameter	Control (Group 1)	Group 2 (26 mg/kg)	Group 3 (39 mg/kg)	Group 4 (78 mg/kg)	F-value	P-value
Catalase (CAT) (U/mg protein)	25.0 ± 2.5	22.0 ± 2.0^a^	18.0 ± 1.8^b^	12.0 ± 1.5^c^	260.86	< 0.0001
Glutathione Peroxidase (GSH) (nmol/mg protein)	40.0 ± 3.0	35.0 ± 2.5^a^	30.0 ± 2.0^b^	20.0 ± 1.8^c^	378.23	< 0.0001
Malondialdehyde (MDA) (nmol/mg protein)	2.5 ± 0.3	3.0 ± 0.4^a^	4.5 ± 0.5^b^	6.0 ± 0.6^c^	507.37	< 0.0001

- A one-way ANOVA was conducted to determine statistical significance (P).

- F-ratio (F) was calculated to show the differences between groups compared to the differences within each group

-N in all groups = 10

- Superscript meanings:

(a): Statistically significant at p ≤ 0.05

(b): Statistically significant at p ≤ 0.02–0.04

(c): Statistically significant at p < 0.001

(ns): Not statistically significant

Changes in serum and urine 8-hydroxy-2′-deoxyguanosine (8-OH-2DG) levels, shown in Table 6, indicate significant oxidative DNA damage in TMX-treated groups. The F-values indicate highly significant differences between control and treated groups, with p-values < 0.0001.

**Table 6:** ANOVA statistical analysis of 8-hydroxy-2′-deoxyguanosine (8-oh-2dg) levels after thiamethoxam exposure.

Parameter	Control (Group 1)	Group 2 (26 mg/kg)	Group 3 (39 mg/kg)	Group 4 (78 mg/kg)	F-value	P-value
Serum 8-OH−2DG in ng/mL, Percentage Increase (%)	1.45 ± 0.10 (N/A)	3.80 ± 0.20^a^ (162.1 %)	6.10 ± 0.40^b^ (320.7 %)	7.67 ± 0.50^c^ (4289 %)	2189.98	< 0.0001
Urine 8-OH−2DG in ng/mL, Percentage Increase (%)	2.10 ± 0.15 (N/A)	4.20 ± 0.25^a^ (100.0 %)	5.90 ± 0.30^b^ (180.9 %)	7.01 ± 0.40^c^ (234.6 %)	1577.32	< 0.0001

- A one-way ANOVA was conducted to determine statistical significance (P).

- F-ratio (F) was calculated to show the differences between groups compared to the differences within each group

-N in all groups = 10

- Superscript meanings:

(a): Statistically significant at p ≤ 0.05

(b): Statistically significant at p ≤ 0.02–0.04

(c): Statistically significant at p < 0.001

(ns): Not statistically significant

Serum 8-OH-2DG levels increased significantly in a dose-dependent manner, with a 162.1 % increase at 26 mg/kg, a 320.7 % increase at 39 mg/kg, and a 428.9 % increase at 78 mg/kg, indicating severe oxidative DNA damage. Urine 8-OH-2DG levels also increased significantly, with a 100.0 % rise at 26 mg/kg, 180.9 % at 39 mg/kg, and 234.6 % at 78 mg/kg, reflecting increased DNA oxidation and excretion of damaged nucleotides.

**Fig. 1:**
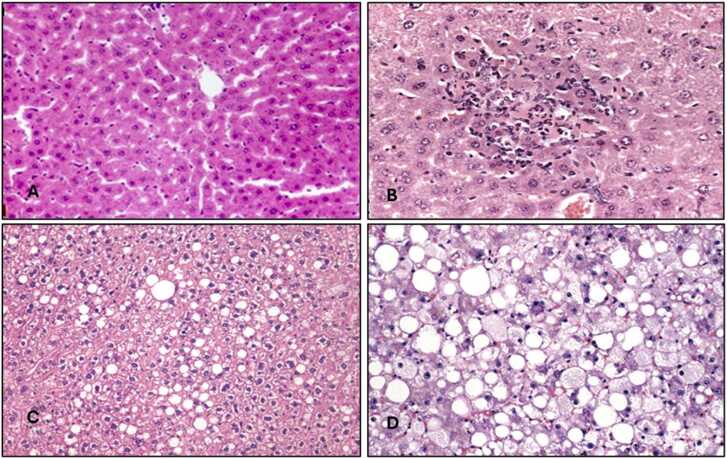
Fig. (1 - A, B, C, D): Histopathology of male albino rat Liver X200 magnification with H & E staining, (1-A: Control) Score 0: Normal, (1-B: TMX 26 mg/kg) Score I: Mild Damage, (1-C: TMX 39 mg/kg) Score II: Moderate Damage, (1-D: TMX 78 mg/kg) Score III: Severe Damage.

**Fig. 2:**
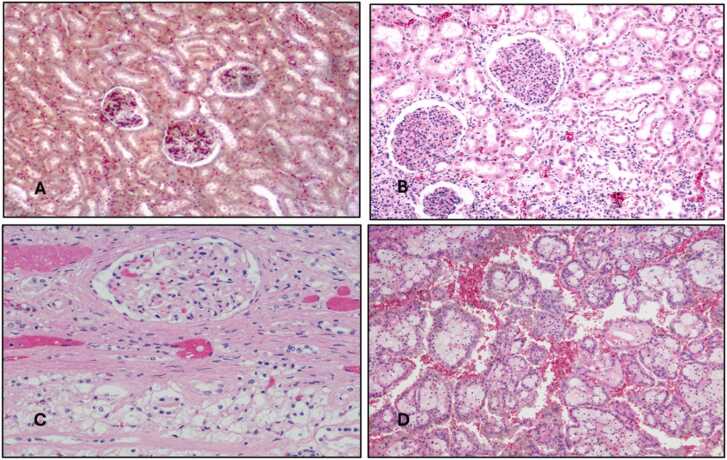
Fig. (2 - A, B, C, D): Histopathology of male albino rat Kidney X200 magnification with H & E staining, (2-A: Control) Score 0: Normal, (2-B: TMX 26 mg/kg) Score I: Mild Damage, (2-C: TMX 39 mg/kg) Score II: Moderate Damage, (2-D: TMX 78 mg/kg) Score III: Severe Damage.

**Fig. 3:**
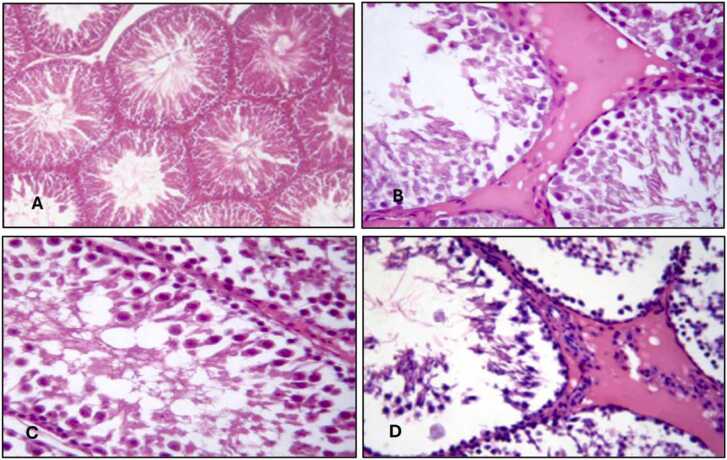
Fig. (3 - A, B, C, D): Histopathology of male albino rat Testis X400 magnification with H & E staining, (3-A: Control) Score 0: Normal, (3-B: TMX 26 mg/kg) Score I: Mild Damage, (3-C: TMX 39 mg/kg) Score II: Moderate Damage, (3-D: TMX 78 mg/kg) Score III: Severe Damage.

**Fig. 4:**
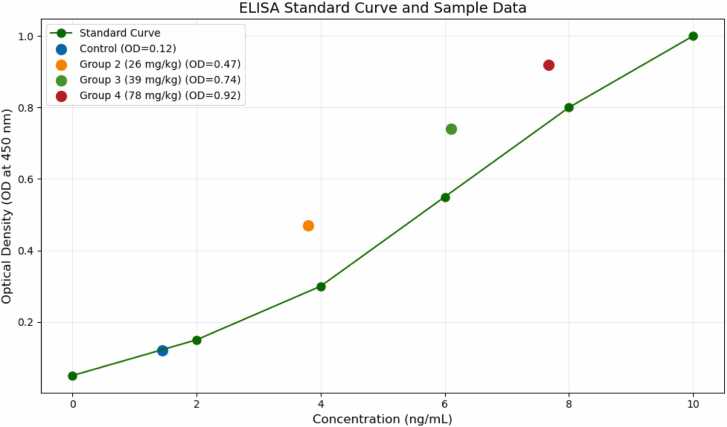
Standard curve of 8-OH-2DG levels (0–10 ng/mL) from ELISA assay in all experimental groups.

Histopathological changes in the liver, kidney, and testicular tissues are detailed in Table 7 and Fig. 1, Fig. 2, Fig. 3. TMX caused dose-dependent tissue damage. Vacuolation and necrosis were observed in the liver, with severe effects in Group 4. Kidney sections showed tubular degeneration and glomerular atrophy, progressing to necrosis at higher doses. Testicular tissues exhibited mild degeneration in Group 2, while Group 4 showed severe degeneration of seminiferous tubules and impaired spermatogenesis. Fig. (4) displays the Standard curve of 8-OH-2DG levels (0–10 ng/mL) from ELISA assay. Data points for control and high-dose groups are highlighted, showing a significant increase in 8-OH-2DG levels in the high-dose group compared to the control.

**Table 7:** Histopathological findings in liver, kidney, and testicular tissues in control and thiamethoxam-treated groups.

Group	Liver	Kidney	Testicular Tissue	Severity (vs. Control)
Control (Group 1)	Normal architecture, no abnormalities	Normal glomeruli and tubules	Normal seminiferous tubules and spermatogenesis	N/A
Group 2 (26 mg/kg)	Mild vacuolation in hepatocytes	Mild tubular degeneration	Mild degeneration of seminiferous tubules	+ (Minimal)
Group 3 (39 mg/kg)	Moderate vacuolation and hepatocyte swelling	Tubular degeneration and glomerular atrophy	Reduced spermatogenesis, interstitial edema	+ + (Moderate)
Group 4 (78 mg/kg)	Severe vacuolation, hepatocyte necrosis	Severe tubular necrosis, interstitial oedema	Severe degeneration of seminiferous tubules	+ ++ (Severe)

-N in all groups = 10

## 5 Discussion

Over the past few decades, increasing evidence has highlighted the harmful impact of environmental toxicants on different body organs and reproductive health. Pesticide toxicity is a significant global issue, especially for species that aren't the intended targets [6], [18]. Pesticides are substances, or mixtures of substances, designed to eliminate unwanted pests like disease-carrying insects, fungi, and weeds. They play a crucial role in boosting food production and supporting the processes of food distribution, storage, and marketing. However, their widespread use raises concerns about the broader environmental and health effects [18].

A key mechanism underlying TMX-induced toxicity is oxidative stress, as demonstrated by decreased antioxidant enzyme activities (CAT, GSH) and increased lipid peroxidation (MDA levels). The significant reduction in CAT and GSH levels in Group IV underscores the depletion of the antioxidant defence system due to excessive ROS generation [8]. The current study provides compelling evidence of the dose-dependent toxic effects of thiamethoxam (TMX) on multiple physiological, biochemical, and histopathological parameters in rats. TMX, a widely used neonicotinoid insecticide, has been associated with significant alterations in organ function, oxidative stress markers, and histological integrity, underscoring its potential risks to non-target species, including mammals [2]. The results of this study indicate that thiamethoxam (TMX) exposure leads to dose-dependent toxic effects on the liver, kidney, and reproductive systems, consistent with findings from previous research [12]. TMX exposure resulted in significant elevations of liver enzymes such as ALT, AST, and ALP, which are key indicators of hepatocellular injury. Histopathological changes, including hepatocellular vacuolation and necrosis, were evident in the high-dose group. These observations align with other researchers [5], [1], who reported that TMX disrupts the oxidative balance in hepatocytes, leading to DNA damage and cellular degeneration in rats. Similarly, Abouelghar et al. found that TMX exposure induces lipid peroxidation in the liver, which correlates with increased oxidative stress markers and decreased antioxidant enzyme activities [2].

Consistent with findings from similar studies, TMX exposure significantly elevated liver enzyme levels (ALT, AST, GGT) and markers of renal dysfunction (urea, creatinine), as observed in this study. Group IV (78 mg/kg) rats demonstrated the most pronounced effects, correlating with severe hepatic and renal histopathological changes, including necrosis and tubular degeneration [5], [22]. Significant increases in urea and creatinine levels were observed, reflecting the compromised renal function. Histological analysis revealed tubular necrosis and glomerular atrophy, consistent with reports by Khaldoun-Oularbi et al., who identified similar renal damage in TMX-exposed rats. Ramanathan S et al. also demonstrated oxidative stress-induced renal injury with elevated malondialdehyde (MDA) levels in TMX-treated rats, supporting our findings [12], [16].

TMX exposure adversely affected reproductive parameters, including testosterone levels, sperm viability, and morphology. Histopathological changes in the testes, such as seminiferous tubule degeneration, were particularly pronounced at higher doses. This agrees with the findings by Zuscikova et al., who highlighted neonicotinoid-induced oxidative stress as a key factor in disrupting spermatogenesis and hormone regulation [24]. Moreover, Abdel-Razik et al. [1] emphasized the role of TMX in inducing oxidative DNA damage in testicular tissues, which could underlie the observed reproductive impairments [1].

Oxidative stress emerges as a central mechanism in TMX toxicity. The observed increase in MDA levels and reduction in antioxidant enzymes (e.g., CAT, GSH) indicate a disrupted redox balance, consistent with Abouelghar et al. [2], who demonstrated that neonicotinoids induce ROS generation and oxidative damage in multiple organs. [2]. The genotoxic potential of TMX was further corroborated by elevated levels of 8-hydroxy-2′-deoxyguanosine (8-OH-2DG), a biomarker of oxidative DNA damage, as previously reported by Abdel-Razik et al. [1].

TMX's impact on reproductive health, evidenced by reduced testosterone levels, sperm parameters, and organ weights (testis, prostate gland, seminal vesicle, and epididymis), highlights its endocrine-disrupting properties. These findings echo previous reports indicating that TMX metabolites can interfere with hormonal regulation and spermatogenesis by generating reactive oxygen species (ROS) and altering cellular signalling pathways (Hamed et al., 2024).

This study builds on earlier findings by comprehensively assessing TMX's systemic toxicity across liver, kidney, and reproductive systems. Unlike prior studies that focused on single organ systems, our research integrates biochemical, oxidative stress, and histopathological analyses to elucidate the dose-dependent effects of TMX. Notably, our results highlight the interconnected nature of organ-specific toxicity, particularly the cascading effects of oxidative stress. The observed nephrotoxicity and hepatotoxicity at subchronic doses underscore the potential health risks for populations with prolonged exposure to TMX, such as agricultural workers. Furthermore, TMX's ability to induce oxidative stress and DNA damage raises concerns about its long-term environmental persistence and impact on aquatic ecosystems.

The findings underscore the need for stricter regulation of TMX usage, particularly in agricultural practices where chronic exposure is likely. Protective interventions, such as antioxidant supplementation (e.g., N-acetylcysteine), may mitigate TMX-induced oxidative damage, as Abdel-Razik et al. suggested [1]. Further research should explore the long-term health implications of low-dose TMX exposure and its potential synergistic effects with other agrochemicals. The exclusion of female models limits our understanding of sex-specific toxicity, particularly concerning hormonal and reproductive parameters. Future studies should incorporate both sexes to provide a comprehensive toxicological profile of TMX. In addition, future research should prioritize evaluating the long-term health effects of chronic low-dose TMX exposure, particularly its potential to induce cumulative oxidative damage. Additionally, exploring antioxidant co-treatment strategies could provide actionable interventions to mitigate TMX's toxic effects.

## 6 Conclusions

The present study provides a comprehensive assessment of thiamethoxam (TMX)-induced toxicity in male albino rats, revealing dose-dependent adverse effects on the liver, kidneys, and reproductive systems. TMX exposure significantly disrupted biochemical markers, oxidative stress parameters, and histopathological integrity, with the highest dose demonstrating the most pronounced toxicity. The findings underscore the oxidative stress-mediated mechanism of TMX toxicity, highlighted by reduced antioxidant enzyme activities (CAT, GSH), increased lipid peroxidation (MDA), and elevated oxidative DNA damage (8-OH-2DG).

This study uniquely integrates biochemical, genetic, and histopathological analyses to elucidate the systemic and reproductive toxicities of TMX. By demonstrating dose-dependent increases in 8-OH-2DG levels and severe histopathological changes in the testes, the research provides novel insights into TMX's genotoxic and endocrine-disrupting potential.

The broader implications of these findings extend to human health and environmental policies. Occupational and environmental exposure to TMX, especially in agricultural settings, could pose significant health risks. These results highlight the urgent need for stricter regulations and mitigation strategies, such as antioxidant interventions, to reduce the toxic effects of TMX.

Future studies should focus on chronic low-dose exposure scenarios, explore sex-specific toxicities, and evaluate protective measures like dietary antioxidants. This research serves as a critical foundation for understanding the multifaceted toxicological impacts of TMX and provides a roadmap for future investigations to ensure safer pesticide practices.

Recommendations: Given the evidence of TMX-induced toxicity, there is a strong need for stricter regulations on its use. Regulatory authorities should consider limiting TMX application in agriculture, particularly in environmentally sensitive areas, and promoting safer alternatives. Additionally, mitigation strategies such as antioxidant supplementation may help alleviate its toxic effects. Future research should focus on evaluating long-term health effects and identifying effective protective measures. Public awareness and policy adjustments should be prioritized to ensure safer pesticide application and reduced environmental contamination.

## Ethical conduct

All procedures performed in this research were per the ethical standards of the Ethical Institutional Review Board Alexandria University, Egypt, and with the 1964 Helsinki Declaration and its later amendments or comparable ethical standards. Adherence to these standards and animal welfare guidelines ensured the welfare of the animals while maintaining the scientific rigour of the research.

## Funding

This research received no specific grant from any funding agency in the public, commercial, or not-for-profit sectors.

## Declaration of Competing Interest

The authors declare that they have no known competing financial interests or personal relationships that could have appeared to influence the work reported in this paper.

## Data Availability

Data will be made available on request.
