# Supplementary material for: Toxicological impact of Thiamethoxam on adult male rats: Histopathological, biochemical, and oxidative DNA damage assessment
Source: Toxicol Rep. 2025 Mar 15;14:101983. doi: 10.1016/j.toxrep.2025.101983 (PMC11979398; doi:10.1016/j.toxrep.2025.101983)
Supplement: Supplementary file 1 — Supplementary material [file mmc1.docx]

**Quantitative Assessment of Tissue Damage in Liver, Kidney, and Testis**

To statistically evaluate the dose-dependent tissue damage induced by Thiamethoxam (TMX), the following quantitative assessments were conducted:

**1. Lesion Counts per Field of View (FOV)**

Tissue damage was assessed by counting the number of lesions in 10 randomly selected high-power fields (HPF, 40× magnification) for each organ.

| **Group** | **Liver Lesions (per 10 HPF)** | **Kidney Lesions (per 10 HPF)** | **Testis Lesions (per 10 HPF)** |
| --- | --- | --- | --- |
| **Control (Group 1)** | 0.0 ± 0.0 | 0.0 ± 0.0 | 0.0 ± 0.0 |
| **26 mg/kg (Group 2)** | 3.2 ± 0.8^a^ | 2.8 ± 0.6^a^ | 4.0 ± 0.9^a^ |
| **39 mg/kg (Group 3)** | 6.5 ± 1.2^b^ | 5.2 ± 1.0^b^ | 8.3 ± 1.5^b^ |
| **78 mg/kg (Group 4)** | 12.1 ± 1.8^c^ | 9.7 ± 1.4^c^ | 15.5 ± 2.0^c^ |

**- Statistical Analysis: One-way ANOVA followed by Tukey’s post hoc test (P < 0.05). Different superscripts (a, b, c) indicate statistically significant differences between groups.**

**2. Percentage of Affected Tissue Area**

Quantification of tissue damage was performed using **ImageJ software** to determine the percentage of the tissue occupied by necrotic or degenerative lesions.

| **Group** | **Liver Damage (%)** | **Kidney Damage (%)** | **Testis Damage (%)** |
| --- | --- | --- | --- |
| **Control (Group 1)** | 0.0 ± 0.0 | 0.0 ± 0.0 | 0.0 ± 0.0 |
| **26 mg/kg (Group 2)** | 8.2 ± 1.4^a^ | 5.6 ± 1.2^a^ | 9.5 ± 1.7^a^ |
| **39 mg/kg (Group 3)** | 15.7 ± 2.2^b^ | 11.4 ± 2.0^b^ | 18.9 ± 2.8^b^ |
| **78 mg/kg (Group 4)** | 27.3 ± 3.5^c^ | 22.8 ± 3.0^c^ | 34.7 ± 4.2^c^ |

**- Statistical Analysis: One-way ANOVA followed by Tukey’s post hoc test (P < 0.05). Different superscripts (a, b, c) indicate statistically significant differences between groups.**

**3. Number of Examined Tissues per Group**

Histopathological evaluation was conducted on 10 randomly selected tissue sections per organ from six animals per group. Two blinded pathologists independently examined each section to minimize observer bias.

**4. Intra-Animal Variation in Pathology**

To assess whether the same animal exhibited similar levels of pathology across different organs, Pearson correlation analysis was performed to evaluate the relationship between lesion severity in the liver, kidney, and testis.

| **Organ Comparison** | **Correlation Coefficient (r)** | **Statistical Significance (P-value)** |
| --- | --- | --- |
| **Liver vs. Kidney** | 0.82 | P < 0.01 |
| **Liver vs. Testis** | 0.65 | P < 0.05 |
| **Kidney vs. Testis** | 0.71 | P < 0.05 |

These results suggest a strong correlation between hepatic and renal lesions, likely due to shared detoxification mechanisms and metabolic burdens. The testicular pathology exhibited a moderate correlation with hepatic and renal lesions, indicating that while systemic toxicity affects all three organs, individual variability in detoxification capacity, oxidative stress resilience, and histopathological thresholds may influence differential organ susceptibility within the same animal.

This intra-animal variation may be related to tissue-specific metabolic activity, antioxidant defences, and cellular repair mechanisms, which may explain why certain animals exhibit higher pathology in one organ compared to another.

**5. Correlation Between Biochemical Markers and Histopathology**

To determine the relationship between biochemical changes and tissue damage, correlation analyses were conducted between biochemical markers (from Tables 1-6) and histopathological findings.

| **Biochemical Marker** | **Organ Damage Correlation (r)** | **Statistical Significance (P-value)** |
| --- | --- | --- |
| **ALT (U/L) vs. Liver Lesions** | 0.85 | P < 0.01 |
| **AST (U/L) vs. Liver Lesions** | 0.80 | P < 0.01 |
| **Creatinine (mg/dL) vs. Kidney Lesions** | 0.78 | P < 0.01 |
| **MDA (nmol/mg) vs. Testis Lesions** | 0.72 | P < 0.05 |

The strong correlations between ALT, AST, liver lesions and creatinine and kidney lesions suggest that biochemical markers reliably reflect tissue pathology. Additionally, the positive correlation between malondialdehyde (MDA) and testicular damage highlights the role of oxidative stress in testicular pathology.

These findings reinforce that biochemical assessments can serve as non-invasive indicators of tissue damage and support the histopathological findings.

**N.B.** This quantitative assessment confirms that Thiamethoxam induced significant dose-dependent histopathological damage in the liver, kidney, and testis. The observed increases in lesion counts and affected tissue area percentages demonstrate a clear relationship between Thiamethoxam exposure and tissue damage severity. Additionally, correlation analyses confirm that biochemical markers are significantly associated with histopathological damage, providing a robust link between functional and structural toxicity.

These findings reinforce the necessity for further molecular and histochemical analyses to elucidate the underlying mechanisms of toxicity and potential recovery pathways.
